# Undulated Step Structure on the (000$\bar{1}$) Facet of Physical Vapor Transport-Grown 4H-SiC Crystals

**DOI:** 10.3390/ma14226816

**Published:** 2021-11-11

**Authors:** Hiroaki Shinya, Masataka Nakano, Noboru Ohtani

**Affiliations:** School of Science and Technology, Kwansei Gakuin University, 2-1 Gakuen, Sanda 669-1337, Hyogo, Japan; cte57141@kwansei.ac.jp (H.S.); dad13517@kwansei.ac.jp (M.N.)

**Keywords:** silicon carbide, facet, step structure, nitrogen doping, step bunching

## Abstract

The step structure on the (000$\bar{1}$)C facet of 4H-SiC boules grown by the physical vapor transport growth method with different nitrogen doping concentrations was examined in various scales, using different types of microscopy, such as differential interference contrast optical microscopy (DICM) and atomic force microscopy (AFM). DICM observations unveiled characteristic macroscopic surface features of the facet dependent on the nitrogen doping concentration. AFM observations revealed the existence of step trains of half unit-cell height (0.5 nm) on the facet and found that their separation was undulated with a characteristic wavelength dependent on the nitrogen doping concentration; the higher the nitrogen concentration, the longer was the undulation wavelength of step separation. Based on these results, we discussed the origin and formation mechanism of the separation-undulated step structure observed on the (000$\bar{1}$)C facet of nitrogen-doped 4H-SiC boules.

## 1. Introduction

Silicon carbide (SiC) single crystals are of great interest in both industrial and scientific contexts. These crystals have been intensively pursued as materials for high performance power semiconductor devices because of their superior physical and electrical properties, such as wide band gap, high dielectric breakdown field, and high thermal conductivity [1]. As for the scientific aspect of SiC single crystals, SiC are noted for their polytypic nature. In SiC, polytypes are described as a number of different one-dimensional ordering sequences without any variation in stoichiometry. They are often referred to as a combination of number and letter, such as 3C, 6H, and 4H; the former denotes the number of Si-C bilayers in the unit cell, and the latter denotes the crystal symmetry (C for cubic, H for hexagonal, and R for rhombohedral). Though well described by previous studies (see for instance [2,3]), their origin is still controversial. The polytypism in SiC crystals is not only a topic of great scientific interest but also a relevant issue for industrial applications of SiC crystals, since foreign polytype crystal inclusions in grown crystals, which often occur during physical vapor transport (PVT) growth of SiC, cause the formation of various types of crystallographic defects in grown crystals [4].

To obtain single-polytype SiC crystals, control over the surface morphology of as-grown crystals is extremely relevant. The well-controlled shape and morphology of the growing crystal surface assure the polytype-preserving crystal growth of SiC. SiC crystal growth through the PVT growth method is believed to proceed via the spiral growth mechanism, and the growing crystal surface of PVT-grown SiC crystals is covered with surface step trains emanating from spiral growth centers. Foreign polytype crystal inclusions in SiC crystals are likely to be caused by instabilities in these step trains, such as step bunching and/or meandering, through the uncontrolled nucleation of foreign polytype crystals on the growing crystal surface. In this respect, a previous report that mentioned that nucleation is likely to occur on the edges of the (000$\bar{1}$) facet of SiC boules is of great interest [5]. The (000$\bar{1}$) facet develops at the center of the growth front of 4H-SiC boules during PVT growth, which is associated with non-facetted regions outside the facet. The facet is a flat plane covered with surface steps emanating from spiral growth centers located on the facet, whereas non-facetted regions are regions covering the crystal growth front other than the facet. It is generally believed that Burton-Cabrera-Frank-type growth kinetics govern crystal growth on the (000$\bar{1}$) facet, resulting in stable, polytype-preserving growth; however, stable growth is occasionally hindered for some reason, and foreign polytype crystals nucleate on the facet.

The step structure on the as-grown surface of SiC crystals has been studied by several research groups [6,7,8,9,10,11,12]. On the (000$\bar{1}$) facet of 4H-SiC crystals grown in the *c*-axis direction, surface steps of half-unit cell height (0.5 nm) were predominantly observed, and they were often bunched into macrosteps of heights of approximately 6–10 nm. Macrosteps formed on the growing crystal surface are believed to play an important role in dislocation deflection and conversion processes [13,14,15,16], which significantly reduce the threading dislocation density in SiC crystals; thus, surface morphology control is also a key issue in obtaining SiC crystals with a low threading dislocation density. However, detailed mechanisms of step instabilities, such as bunching (macrostep formation) and meandering on the growing crystal surface of SiC, are still not well understood; and a method through which to control the surface morphology of SiC crystals and achieve stable polytype-preserving crystal growth with a low dislocation density is yet to be determined.

This article investigates the surface morphology of the (000$\bar{1}$)C facet of 4H-SiC boules, particularly the nitrogen doping concentration dependence of the step structure on the (000$\bar{1}$) facet. The (000$\bar{1}$) facet exhibits a significant difference in the step separation between the central and edge regions of the facet [11]; thus, different step energetics and kinetics govern the step morphology in the two regions. It was found that the step trains observed in the edge region of the (000$\bar{1}$) facet of the 4H-SiC boules showed a separation undulation, i.e., step bunching, and the wavelength of the undulation was dependent on the nitrogen doping concentration in the crystals; whereas in the central region of the facet, the characteristic meandering behaviors of the surface steps were revealed. Based on these results, we discuss the cause and formation mechanism of the nitrogen concentration-dependent step structure observed on the (000$\bar{1}$) facet of 4H-SiC boules.

## 2. Experimental Procedure

One- or two-inch (25.4 or 50.8 mm)-diameter 4H-SiC single-crystal boules were grown on an on-axis (000$\bar{1}$) 4H-SiC seed crystal through the PVT growth method. The typical growth temperature was approximately 2300–2400 °C, and the argon gas pressure was maintained between 1.0 and 2.0 kPa during growth. The crystallographic orientation of the seed crystal was determined using X-ray diffraction. The grown boules were nitrogen-doped, and they contained nitrogen donors in the mid-10^17^ (nominally undoped), mid-10^18^ (conventionally doped) or mid-10^19^ cm^−3^ (heavily doped) range. They are referred to as boules “A”, “B”, and “C”, respectively, in this paper. The macroscopic (millimeter and centimeter scale) surface morphologies of the (000$\bar{1}$) facet of the 4H-SiC boules were examined by differential interference contrast optical microscopy (DICM) (Olympus MX51, Tokyo, Japan) and confocal laser scanning microscopy (CLSM) (Keyence VK-9700, Osaka, Japan). DICM images were obtained with ×5, ×10, and ×20 objective lenses, and millimeter- and centimeter-size photographs were made up of a patchwork of these images. CLSM was used to measure the local inclination of the facet surface tilted from the (000$\bar{1}$) basal plane. The CLSM images were obtained with a 658-nm laser diode, and the local inclination was obtained by measuring the height difference across the observed area. The surface morphology assessments with micrometer- and nanometer-scale resolutions were performed by using low-voltage scanning electron microscopy (LVSEM) (Carl Zeiss Gemini-Supra 40, Oberkochen, Germany) and atomic force microscopy (AFM) (Bruker Dimension Icon, Madison, USA). AFM images were obtained at a 512 × 512-point resolution over 0.9-μm, 10-μm, 20-μm, 25-μm, 30-μm or 50-μm square areas through PeakForce tapping mode measurements, using a silicon nitride cantilever. LVSEM observations were performed with an acceleration voltage of 1 kV and a working distance of ~4 mm.

## 3. Results and Discussion

**Figure 1:**
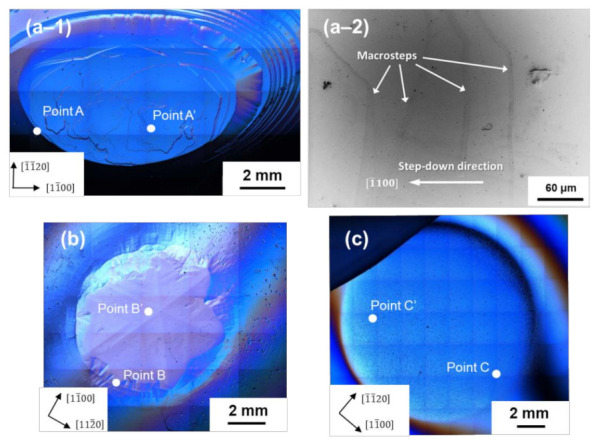
DICM images of the (000$\bar{1}$) facet of two-inch diameter (**a–1**) nominally undoped (boule A, N concentration: mid-10^17^ cm^−3^), (**b**) conventionally nitrogen-doped (sample B, N concentration: mid-10^18^ cm^−3^), and (**c**) heavily nitrogen-doped 4H-SiC boules (sample C, N concentration: mid-10^19^ cm^−3^). AFM observations were conducted at Points A, B, C, A’, B’, and C’, as shown in the images. The former three points were located near the edge of the (000$\bar{1}$ ) facet, whereas the latter were located near spiral growth centers on the facet. (**a–2**) LVSEM image of macrosteps observed on the facet of boule A.

Figure 1 shows the DICM images of the surface morphology of the (000$\bar{1}$) facet of the two-inch diameter (a–1) nominally undoped (boule A), (b) conventionally nitrogen-doped (boule B), and (c) heavily nitrogen-doped 4H-SiC boules (boule C) examined in this study. The figure reveals that the growth front of the boules comprised two distinct morphological regions: the (000$\bar{1}$) facet and the non-facetted region.

**Figure 2:**
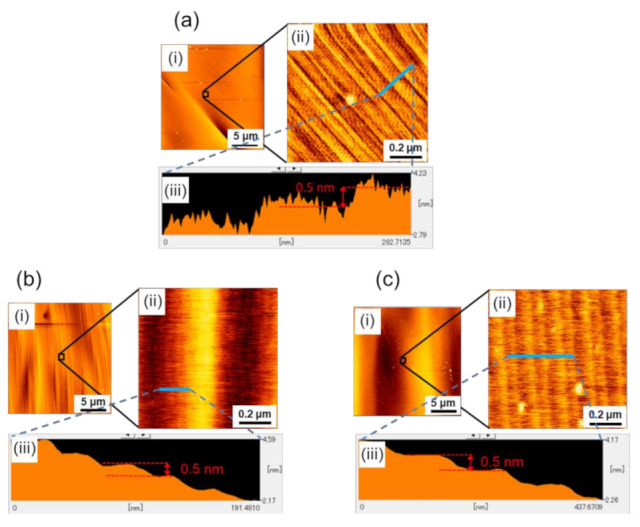
AFM images at (**a**) Point A, (**b**) Point B, and (**c**) Point C near the edge of the (000$\bar{1}$) facet of boules A, B, and C, respectively. In each figure, (**i**) wide-area AFM image (20-μm square area), (**ii**) small-area image (0.9-μm square area), and (**iii**) height profile are shown, where the small-area AFM image was taken from the area indicated by an open square in (**i**), and the height profile was taken along the blue line indicated in (**ii**).

On the (000$\bar{1}$) facet of the nominally undoped boule (boule A), large macrosteps of height of sub-μm range were observed, and the macrosteps tended to be arranged in petal shapes with a hexagonal symmetry. The height of the macrosteps became larger as their location approached the edge of the (000$\bar{1}$) facet, as confirmed by LVSEM (see Figure 1(a–2)), and a giant slope was found at the boundary between the (000$\bar{1}$) facet and the non-facetted region. Between the macrosteps, fairly flat, almost featureless terraces extended. For sample B, the (000$\bar{1}$) facet also appeared petal-shaped, but no macrosteps of sub-μm height were observed on the facet. The boundary between the (000$\bar{1}$) facet and the non-facetted region was relatively smooth, and an intermediate region was present between the facet and the non-facetted region [11]. The facet surface of boule B showed hexagonal symmetry, comprising six vicinal (000$\bar{1}$) surfaces slightly tilted toward <1$\bar{1}$00> directions, i.e., prism plane directions, accompanied by six crystallographically-equivalent ridges extending along the <11$\bar{2}$0> directions, and the vicinal (000$\bar{1}$) surface exhibited a slightly undulated morphology. On the other hand, the (000$\bar{1}$) facet surface of the heavily nitrogen-doped 4H-SiC boule (boule C) was extremely flat. The surface was almost featureless (with no macrosteps and undulations), and the boundary between the facet and the non-facetted region was obscure. As seen in the figure, a number of very small dots was observed on the almost featureless facet surface of sample C. They are hillocks nucleated during the cooling stage of PVT growth process [17]. Their formation only occurs on the (000$\bar{1}$) facet of heavily nitrogen-doped 4H-SiC boules and is thought to be related to the stacking fault formation in heavily nitrogen-doped 4H-SiC crystals [17].

To reveal the microscopic origin of the observed macroscopic surface morphologies on the (000$\bar{1}$) facet of 4H-SiC boules, we conducted AFM observations of the step structure on the facet. First, we examined the step structure in the edge region of the (000$\bar{1}$) facet of three differently nitrogen-doped 4H-SiC boules (boules A, B, and C). The locations of the AFM observations are indicated as Points A, B, and C in Figure 1 for boules A, B, and C, respectively. The locations were determined in such a way that the tilt angles from the basal plane of each location, which was measured by CLSM, were almost the same (0.3°–0.8°). Figure 2a–c show the results of the AFM observations at Points A, B, and C, respectively. In each figure, (i) a wide-area AFM image (20-μm square area), (ii) a small-area image (0.9-μm square area), and (iii) the height profile are shown, where the small area AFM image was taken from the area indicated by an open square in (i), and the height profile was taken along the blue line indicated in (ii). As seen in the figures, microscopically, the facet surfaces of all three boules were covered with steps of half unit-cell height (0.5 nm), but their configurations were slightly different; the white spots observed in some of the AFM images are particles of, what was presumed to be dust. Difference was observed in the non-uniformity of the step separation (terrace width); there was a characteristic undulation of step separation for each boule. Examples of such an undulation of step separation are clearly demonstrated in Figure 2(bii,ci), where periodically corrugated structures of the step trains were observed. The small area AFM images (Figure 2(bii,cii)) revealed that these corrugated structures consisted of half unit-cell height steps and were caused by their separation modulation.

**Figure 3:**
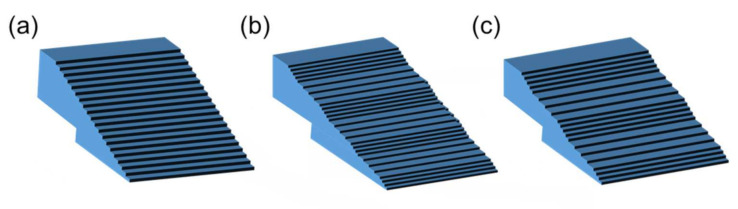
Schematic figures of the separation-undulated step structures on a vicinal crystal surface, where the undulation wavelength of step separation (terrace width) increases from (**a**) to (**b**) to (**c**).

As seen in the figures, the wavelength of the undulation was different among the boules, indicating that the wavelength of the undulation was dependent on the doping concentration of the nitrogen donors in the boules. Table 1 summarizes the nitrogen doping concentration dependence of the undulation wavelength of the step separation, and Figure 3 shows schematic figures of the separation-undulated step trains on a vicinal surface, where the undulation wavelength of step separation (terrace width) increases from Figure 3a–c.

We could not observe any undulation in the step separation on the (000$\bar{1}$) facet edge of boule A (the nominally undoped boule), to which we assigned an undulation wavelength of <70 nm, comparable to or less than the step separation. As shown in the table, as the nitrogen doping concentration increased (from boule A to boule B to boule C), the undulation wavelength significantly increased.

**Figure 4:**
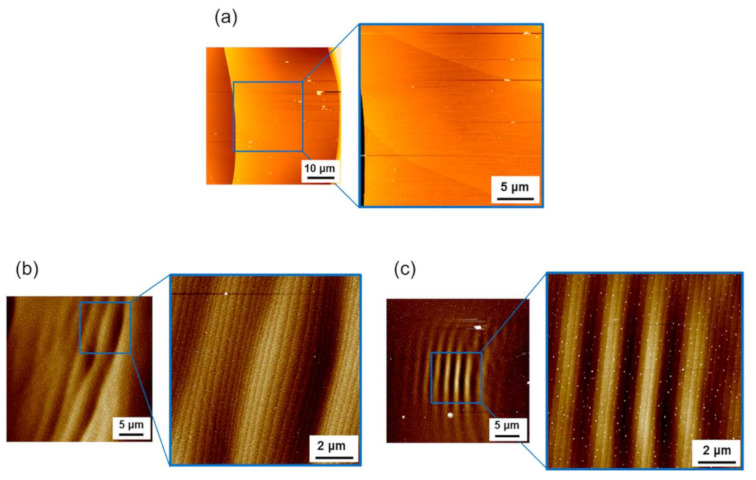
AFM images of the step structures observed at (**a**) Point A’, (**b**) Point B’, and (**c**) Point C’ in the central region (near a spiral growth center) of the (000$\bar{1}$) facet of boules A, B, and C, respectively, shown in Figure 1. In each figure, a set of wide-area AFM images (50-μm square or 30-μm square area) and small-area images (25-μm square or 10-μm square area) are shown; the location of the small-area AFM image is indicated by a blue-line open square shown in the wide area image.

To shed more light on the observed undulated step structure near the facet edge, we examined the step structure in the central region (near a spiral growth center) of the (000$\bar{1}$) facet. Figure 4a–c show the AFM images of the step structures observed at Points A’, B’, and C’ on the facets of boules A, B, and C, respectively, shown in Figure 1. In each figure, a set of wide-area AFM images (50-μm square or 30-μm square area) and small-area images (25-μm square or 10-μm square area) are shown; the location of the small-area AFM image is indicated by a blue-line open square shown in the wide-area image. As shown in the figures, the facet surface in the central region was also covered with half unit-cell height step trains, which were also undulated at a certain wavelength. The step separation and the undulation wavelength of the step separation in the central region of the (000$\bar{1}$) facet of boules A, B, and C are summarized in Table 1, where the wavelength of boule A could not be measured, since the step separation was significantly large. For boule B, undulated step structures were observed in very limited areas in the central region of the facet. We carefully examined the central region of the facet of boule B and found that undulated step structures were rarely observed in the region. As seen in the table, the undulation wavelength of the step separation was very similar for boules B and C and appeared not to be dependent on the nitrogen doping concentration.

As Yamaguchi et al. [11] pointed out, on the (000$\bar{1}$) facet of 4H-SiC crystals, the step separation is larger in the central region and becomes smaller toward the facet edge, according to the variation of undercooling over the facet. This trend was also observed for boules A, B, and C examined in this study, as shown in Table 1. They contained different concentrations of nitrogen donors but exhibited a similar variation of step separation from the central to the edge regions of the (000$\bar{1}$) facet. The step separation was similar among boules A, B, and C except for the central region of the facet for boule A (N: mid-10^17^ cm^−3^), which showed a step separation approximately 30 times larger than those observed for boules B and C. The large step separation in the central region of boule A would have resulted from the formation of macrosteps of sub-μm height on the facet.

In Table 1, we also present the meandering wavelength of the half unit-cell height steps observed in the central region of the (000$\bar{1}$) facet of boules A, B, and C. On the facet of boule C, the half unit-cell height steps were fairly straight; thus, the meandering of the surface steps was assumed to be fully suppressed in the central region of boule C.

We assume that the observed undulation of step separation is a precursor state of step bunching on a vicinal surface [18]. Step bunching is generally thought to have a kinematical origin, and the most widely accepted mechanism is the asymmetric incorporation kinetics of adatoms at surface steps. A well-known form of asymmetric incorporation kinetics is the so-called Ehrlich-Schwoebel (ES) effect, which assumes the preferential incorporation of adatoms to steps from the lower terrace [19,20]; a large kinetic barrier is assumed for adatoms to descend the step edge to the lower terrace. This form of asymmetric kinetics, however, causes step bunching only for negative crystal growth, i.e., the etching of crystal. Hence, to induce step bunching for crystal growth, an inverse ES effect needs to be considered [21,22,23], in which a larger kinetic barrier exists for the adatom incorporation from the lower terrace; thus, adatoms are preferentially incorporated to steps from the upper terrace.

The inverse ES effect has already been proposed to explain the coexistence of step bunching and meandering on the 4H-SiC(0001) surface [21]. However, its validity for the 4H-SiC(000$\bar{1}$) surface is yet to be proven. In this regard, an important requirement to justify the model is that the model can explain the experimental results regarding the undulation wavelength of step separation on the 4H-SiC (000$\bar{1}$) facet. One important conclusion for the observed undulation of step separation (step bunching) on the (000$\bar{1}$) facet of nitrogen-doped 4H-SiC boules is that the undulation wavelength became longer when the nitrogen doping concentration increased.

As described earlier in this section, the nitrogen doping concentration also affects the line profile of surface steps. The AFM images shown in Figure 4 indicate that the meandering wavelength of the half unit-cell height steps became shorter as the nitrogen doping concentration increased, except for in boule C; we discuss the reason why fairly straight steps were observed on the (000$\bar{1}$) facet of boule C below.

Step meandering generally occurs through the competition between the kinematical (destabilizing) and energetic (stabilizing) effects on the step morphology [24]; the former induces step meandering, whereas the latter stabilizes the straight-line morphology of the surface steps. Here, an important parameter for the energetic effect is the line tension of the steps, i.e., the step stiffness. The step stiffness is the measure of resistance against the kinematical driving force for step meandering and determines the meandering wavelength of the surface steps [24]; the larger the step stiffness, the longer the meandering wavelength. Hence, the results of the AFM observations shown in Figure 4 indicate that by some mechanism, nitrogen doping of 4H-SiC crystals reduces the step stiffness on the (000$\bar{1}$) surface, making the meandering wavelength shorter as the nitrogen doping concentration increases. The macroscopic facet morphologies observed for boules A, B, and C lend support to this conclusion. As shown in Figure 1, the facet morphology of the nitrogen-doped 4H-SiC crystals became more isotropic and smoother as the nitrogen doping concentration increased, indicating that energetics (step stiffness), which usually featured a preferred step flow direction reflecting the crystal symmetry, did not greatly influence the facet morphology at a high nitrogen doping concentration.

Usually, a small step stiffness results in a largely meandering step morphology on the growing crystal surface; however, the half unit-cell height steps observed on the (000$\bar{1}$) facet of boule C, which were assumed to have a small step stiffness, showed a fairly straight step morphology. This was due to the enhanced diffusion length of the adatoms on the (000$\bar{1}$) facet of boule C. As we discuss later in this study, heavy nitrogen doping modified the bonding structure of the 4H-SiC (000$\bar{1}$) surface, leading to the enhancement of the diffusion length of the surface adatoms on the growing crystal surface and, consequently, suppressing the step meandering in spite of the small step stiffness [24].

The influence of the step stiffness on the step bunching behavior was investigated by Sato and Uwaha [25]. They theoretically investigated the instability of step trains during negative crystal growth (sublimation), assuming an ES-type asymmetric incorporation kinetics of adatoms to the steps. Their calculation took into consideration the step stiffness through the step repulsive interaction. A larger step stiffness gives rise to a larger elastic repulsion interaction between surface steps. They successfully demonstrated step bunching (undulation of step separation) with an asymmetric incorporation kinetics, and their results indicated that the larger the step repulsive interaction, the longer the undulation wavelength. This trend is completely opposite to our experimental results, according to which the undulation wavelength became longer when the step interaction (step stiffness) was reduced by nitrogen doping.

**Figure 5:**
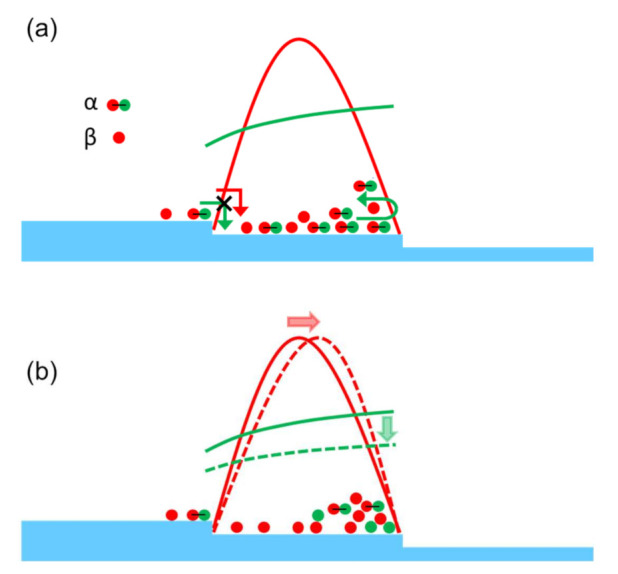
Schematic illustration of the two-particle model proposed by Pimpinelli et al. [26,27,28,29,30,31]. In the figure, the precursor molecules α are depicted as a combination of red and green spheres, while the adatoms β are depicted as red spheres, which are generated through the cracking of the precursor molecules α on the growing crystal surface. (**a**) shows the distributions of the precursor molecules α and adatoms β without taking into account the cracking of the precursor molecules, whereas (**b**) shows the distributions within it.

To address this problem, we need to consider another mechanism that causes step bunching during crystal growth. A plausible mechanism is the so-called “two-particle” model proposed by Pimpinelli et al. [26,27,28,29,30,31], which is based on the coupling between the surface densities of diffusing precursor molecules α and of adatoms β, in the presence of the ES barrier at the step edges. The adatoms β are constituents of the crystal and contribute directly to the crystal growth. The precursor molecules α dissociate (crack) on the growing crystal surface, yielding the adsorbed adatoms β on the surface, which contribute indirectly to crystal growth. This is the situation occurring during the PVT growth of SiC; crystal growth occurs through the cracking of precursor molecules such as SiC_2_ [32]. Therefore, the model would fit the PVT growth of SiC. Pimpinelli et al. showed that the presence of the ES barrier for the precursor molecules α causes adatom flows in the down-step direction, leading to the conversion of equidistant step trains into separation-undulated step trains (step bunching), even when the adatoms β possess a larger kinetic barrier to descend the steps to the lower terrace compared to when ascending the steps. The two-particle model is schematically illustrated in Figure 5, where the precursor molecules α are depicted as a combination of red and green spheres, whereas the adatoms β are depicted as red spheres, which are generated through the cracking of the precursor molecules α on the growing crystal surface.

If the adatoms do not show asymmetric kinetics for their incorporation to the steps, their distribution is symmetrical on the terraces, as in the distribution schematically illustrated by the red solid line in Figure 5a. The two-particle model assumes the presence of a large ES barrier for the precursor molecules α to descend the steps from the upper terrace, while they could ascend the steps from the lower terrace relatively easily. This asymmetrical form of migration kinetics significantly modifies the distribution of the precursor molecules on the terrace, and the molecules are preferentially found near the terrace edge in the down-step direction, as schematically illustrated by the green solid line in Figure 5a. In the two-particle model, the adatoms β, which constitute the crystal, are provided by the cracking of the precursor molecules; thus, the asymmetrical distribution of the precursor molecules induces the asymmetrical distribution of the adatoms β. The distributions of the precursor molecules and adatoms on the terrace with consideration of the cracking of the precursor molecules are indicated by the red and green dashed lines, respectively, in Figure 5b. The resulting asymmetric distribution of the adatoms (red dashed line in Figure 5b) induces a nominal flow of adatoms from the upper to the lower terrace, even when they feature a larger kinetic barrier for incorporation to the steps from the upper terrace compared to the incorporation from the lower terrace [26,27,28,29,30,31]; this nominal adatom flow toward the down-step direction induces step bunching during crystal growth.

The two-particle model fits the PVT growth of 4H-SiC crystals and could explain the step bunching observed on the (000$\bar{1}$) facet of the crystals. Pimpinelli et al. also showed that in the two-particle model, the step stiffness modifies the step bunching behavior. An important conclusion in their study is that the undulation wavelength of the step separation depends on the magnitude of the step stiffness; the weaker the step stiffness, the longer the undulation wavelength. This behavior is in accordance with our experimental results, summarized in Table 1, according to which the undulation wavelength increases with the increase in the nitrogen doping concentration.

**Figure 6:**
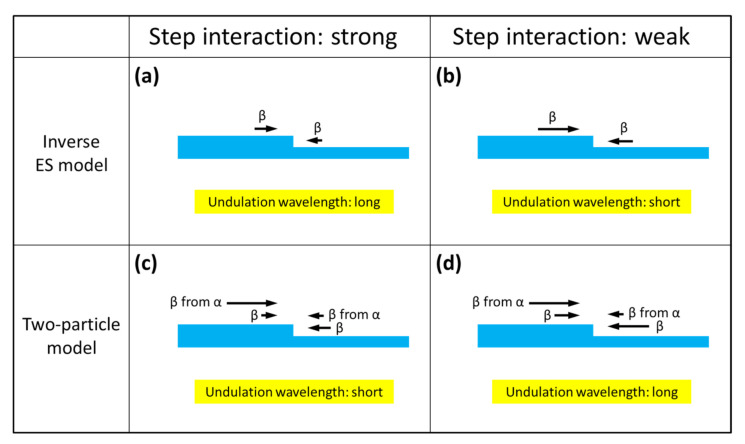
Schematic figures illustrating the effect of the step repulsive interaction on the undulation wavelength of step separation for the inverse ES model and the two-particle model. In the figure, the direction and magnitude of the adatom flows toward the steps on a vicinal crystal surface are indicated by the black arrows, and in each section of the figure, the influence of the step repulsive interaction on the adatom flows is schematically illustrated; the adatom flows toward the steps, which express the amount of adatoms incorporated into the steps from the upper and lower terraces, are reduced by the step repulsive interaction. (**a**) the inverse ES model with a strong step repulsive interaction, (**b**) the inverse ES model with a weak step repulsive interaction, (**c**) the two-particle model with a strong step repulsive interaction, and (**d**) the two-particle model with a weak step repulsive interaction.

Figure 6 schematically represents the effect of the step repulsive interaction (step stiffness) on the undulation wavelength of step separation for the two models, i.e., the inverse ES model and the two-particle model.

In the figure, the direction and magnitude of the adatom flows toward the steps on a vicinal crystal surface are indicated by the black arrows, and in each section of the figure, the influence of the step repulsive interaction on the adatom flows is schematically illustrated; the adatom flows toward the steps, which express the amount of adatoms incorporated into the steps from the upper and lower terraces, are reduced by the step repulsive interaction. In the inverse ES model, the adatom flows from the upper terraces toward the steps are always larger than those from the lower terraces (Figure 6a), and this asymmetry in the adatom flow is a kinematical driving force for step bunching. Therefore, when the step interaction (step stiffness) decreases, as the nitrogen doping concentration increases, the asymmetry (difference in the adatom flows from the upper and lower terraces) becomes larger, as schematically shown in Figure 6b, making the kinematical driving force for the step bunching larger, resulting in a shorter undulation wavelength of step separation.

By contrast, in the two-particle model, the adatom flows from the upper terraces to steps never exceed those from the lower terraces, due to the ES barrier (Figure 6c). In the model, however, the additional adatom flows produced by the cracking of the precursor molecules α are taken into account, making the total adatom flows from the upper terraces larger than those from the lower terraces, thus leading to step bunching during crystal growth (Figure 6c). When the step interaction decreases in this model, the adatom flows from both terraces are enhanced, as is the case with the inverse ES model; however, the additional adatom flows produced by the cracking of the precursor molecules remain almost constant, since the distribution of the precursor molecules on the terraces is not greatly affected by the step interaction. Therefore, the difference in the adatom flows from the upper and lower terraces is reduced (Figure 6d), giving rise to a longer undulation wavelength of step separation, when the step interaction becomes weaker as the nitrogen doping concentration increases.

As shown in Table 1, the undulation wavelength of the step separation in the central region of the (000$\bar{1}$) facet was nearly independent on the nitrogen doping concentration, whereas the dependence was prominent in the edge region. We assume that the largely different step separation in the two regions played a key role in this phenomenon. Firstly, the larger step separation significantly reduces the elastic step interaction, which makes the influence of nitrogen doping less effective, resulting in the undulation wavelength independent of the nitrogen doping concentration. Secondly, as already described, step bunching is generally driven by kinematical interaction between steps via adatom diffusion on the crystal surface; thus, a larger step separation tends to suppress the development of the undulated step structure. In fact, undulation of the step separation was never or rarely observed in the central region of the (000$\bar{1}$) facet of boules A and B. By contrast, for boule C, the undulation of the step separation well developed even in the central region of the (000$\bar{1}$) facet. This was due to the enhanced diffusion length of the adatoms on the (000$\bar{1}$) surfaces of the heavily nitrogen-doped 4H-SiC crystals through the increased effective C/Si ratio due to heavy nitrogen doping [33,34]. Borysiuk et al. [34] reported, based on density functional theory (DFT) calculations, that when the 4H-SiC (000$\bar{1}$) surface features excess carbon atoms, they tend to convert a topmost carbon layer to sp^2^ bonded configuration, which makes the (000$\bar{1}$) surface less active in terms of adatoms and enhances their diffusion length on the surface. Nitrogen atoms, which substitute the carbon site in SiC crystals, play a similar role at the growing crystal surface; heavy nitrogen doping enriches the growing crystal surface with nitrogen atoms, which induces the sp^2^ bonded configuration at the growing crystal surface and enhances the adatom diffusion length on the surface during the PVT growth of heavily nitrogen-doped 4H-SiC crystals.

## 4. Conclusions

We investigated the step structure on the (000$\bar{1}$) facet of 4H-SiC single crystal boules grown using the PVT growth method with different nitrogen doping concentrations, using DICM, CLSM, LVSEM, and AFM. DICM revealed characteristic macroscopic features of the facet, which largely depended on the nitrogen doping concentration; a 4H-SiC boule with a nitrogen concentration of mid-10^17^ cm^−3^ demonstrated large macrosteps of sub-μm height on the facet with a hexagonal symmetry, whereas a boule with a nitrogen concentration of mid-10^19^ cm^−3^ showed a very smooth and isotropic facet morphology. AFM observations unveiled fairly straight step trains in the edge region of the (000$\bar{1}$) facet and found that they formed undulated structures in their separation, and the undulation wavelength of step separation was largely dependent on the nitrogen doping concentration. It was found that the nitrogen doping concentration also affected the meandering behavior of the surface steps of the half unit-cell height in the central region of the facet, and the meandering wavelength became shorter as the nitrogen doping concentration in 4H-SiC boules increased. Based on these results, we discussed the origin and formation mechanism of the undulated step structures observed on the (000$\bar{1}$) facet and suggested that the repulsive step interaction on the facet plays an important role in the observed nitrogen concentration-dependent undulated step structure.

## Figures and Tables

**Table 1:** Nitrogen doping concentration dependence of the average step separation between half-unit cell height steps and the undulation wavelength of step separation in the central and the edge regions of the (000$\bar{1}$ ) facet, and the meandering wavelength of half-unit cell height steps in the central region of the facet of boule A (N: mid-10^17^ cm^−3^), boule B (N: mid-10^18^ cm^−3^), and boule C (N: mid-10^19^ cm^−3^).

Sample	Location	Step Separation	Undulation Wavelength of Step Separation	Meandering Wavelength of Step
boule A	Near facet center	18 μm	NA	100–150 μm
	Near facet edge	70–90 nm	Less than 70 nm	
boule B	Near facet center	300–600 nm	3.2 μm	16–22 μm
	Near facet edge	30–60 nm	0.4 μm	
boule C	Near facet center	490–510 nm	2.2 μm	NA
	Near facet edge	80–100 nm	10.0 μm	

## References

[1] Kimoto T., Cooper J.A. (2014). Fundamentals of Silicon Carbide Technology: Growth, Characterization, Devices and Applications.

[2] Baronnet A. (1978). Some aspects of polytypism in crystals. Prog. Cryst. Growth Charact..

[3] Trigunayat G.C. (1991). A survey of the phenomenon of polytypism in crystals. Solid State Ion..

[4] Chaussende D., Ohtani N., Fornari R. (2018). Silicon carbide. Single Crystals of Electronic Materials: Growth and Properties.

[5] Schmitt E., Straubinger T., Rasp M., Vogel M., Wohlfart A. (2008). Polytype stability and defects in differently doped bulk SiC. J. Cryst. Growth.

[6] Ohtani N., Katsuno M., Takahashi J., Yashiro H., Kanaya M. (1998). Stepped structure on the {0001} facet plane of α-SiC. Surf. Sci. Lett..

[7] Ohtani N., Katsuno M., Takahashi J., Yashiro H., Kanaya M. (1999). Evolution of macrosteps on 6H-SiC(0001): Impurity-induced morphological instability of step trains. Phys. Rev. B.

[8] Herro Z.G., Epelbaum B.M., Bickermann M., Masri P., Winnacker A. (2004). Effective increase of single-crystalline yield during PVT growth of SiC by tailoring of temperature gradient. J. Cryst. Growth.

[9] Guo H.-J., Huang W., Liu X., Gao P., Zhuo S.-Y., Xin J., Yan C.-F., Zheng Y.-Q., Yang J.-H., Shi E.-W. (2014). Analysis of polytype stability in PVT grown silicon carbide single crystal using competitive lattice model Monte Carlo simulations. AIP Adv..

[10] Liu C., Chen X., Peng T., Wang B., Wang W., Wang G. (2014). Step flow and polytype transformation in growth of 4H-SiC crystals. J. Cryst. Growth.

[11] Yamaguchi T., Ohtomo K., Sato S., Ohtani N., Katsuno M., Fujimoto T., Sato S., Tsuge H., Yano T. (2015). Surface morphology and step instability on the (000$\bar{1}$)C facet of physical vapor transport-grown 4H–SiC single crystal boules. J. Cryst. Growth.

[12] Arzig M., Salamon M., Hsiao T.C., Uhlmann N., Wellmann P.J. (2020). Influence of the growth interface shape on the defect characteristics in the facet region of 4H-SiC single crystals. J. Cryst. Growth.

[13] Dudley M., Wu F., Wang H., Byrappa S., Raghothamachar B., Choi G., Sun S., Sanchez E.K., Hansen D., Drachev R. (2011). Stacking faults created by the combined deflection of threading dislocations of Burgers vector c and c+a during the physical vapor transport growth of 4H–SiC. Appl. Phys. Lett..

[14] Yamamoto Y., Harada S., Seki K., Horio A., Mitsuhashi T., Ujihara T. (2012). High-efficiency conversion of threading screw dislocations in 4H-SiC by solution growth. Appl. Phys. Express.

[15] Harada S., Yamamoto Y., Xiao S., Koike D., Mutoh T., Murayama K., Aoyagi K., Sakai T., Tagawa M., Ujihara T. (2015). Dislocation conversion during SiC solution growth for high-quality crystals. Mater. Sci. Forum.

[16] Komatsu N., Mitani T., Hayashi Y., Suo H., Kato T., Okumura H. (2019). Application of defect conversion layer by solution growth for reduction of TSDs in 4H-SiC bulk crystals by PVT growth. Mater. Sci. Forum.

[17] Ohtomo K., Matsumoto N., Ashida K., Kaneko T., Ohtani N., Katsuno M., Sato S., Tsuge H., Fujimoto T. (2017). Formation of basal plane stacking faults on the (000$\bar{1}$) facet of heavily nitrogen-doped 4H-SiC single crystals during physical vapor transport growth. J. Cryst. Growth.

[18] Jeong H.-C., Williams E.D. (1999). Steps on surfaces: Experiment and theory. Surf. Sci. Reports.

[19] Ehrlich G., Hudda F.G. (1966). Atomic view of surface self-diffusion: Tungsten on tungsten. J. Chem. Phys..

[20] Schwoebel R.L. (1968). Step motion on crystal surfaces. II. J. Appl. Phys..

[21] Krzyżewski F., Załuska-Kotur M.A. (2014). Coexistence of bunching and meandering instability in simulated growth of 4H-SiC(0001) surface. J. Appl. Phys..

[22] Bellmann K., Pohl U.W., Kuhn C., Wernicke T., Kneissl M. (2017). Controlling the morphology transition between step-flow growth and step-bunching growth. J. Cryst. Growth.

[23] Seino K., Oshiyama A. (2021). Microscopic mechanism of adatom diffusion on stepped SiC surfaces revealed by first-principles calculations. Appl. Sur. Sci..

[24] Bales G.S., Zangwill A. (1990). Morphological instability of a terrace edge during step-flow growth. Phys. Rev. B.

[25] Sato M., Uwaha M. (1995). Morphological instability caused by asymmetry in step kinetics. Phys. Rev. B.

[26] Pimpinelli A., Videcoq A. (2000). Novel mechanism for the onset of morphological instabilities during chemical vapour epitaxial growth. Surf. Sci. Lett..

[27] Vladimirova M., Pimpinelli A., Videcoq A. (2000). A new model of morphological instabilities during epitaxial growth: From step bunching to mounds formation. J. Cryst. Growth.

[28] Pimpinelli A., Videcoq A., Vladimirova M. (2001). Kinetic surface patterning in two-particle models of epitaxial growth. Appl. Surf. Sci..

[29] Videcoq A., Vladimirova M., Pimpinelli A. (2001). Kinetic surface structuring during homoepitaxy of GaAs (110): A model study. Appl. Surf. Sci..

[30] Videcoq A., Vladimirova M., Pimpinelli A. (2001). Kinetic Monte Carlo study of the terrace width distribution during step bunching in homoepitaxial growth. Appl. Surf. Sci..

[31] Pimpinelli A., Cadoret R., Gil-Lafon E., Napierala J., Trassoudaine A. (2003). Two-particle surface diffusion-reaction models of vapour-phase epitaxial growth on vicinal surfaces. J. Cryst. Growth.

[32] Drowart J., De Maria G., Inghram M.G. (1958). Thermodynamic study of SiC utilizing a mass spectrometer. J. Chem. Phys..

[33] Matsumoto N., Shinya H., Ashida K., Kaneko T., Ohtani N., Katsuno M., Tsuge T., Sato S., Fujimoto T. (2018). Investigation of run-to-run fluctuation in growth conditions of physical vapor transport growth of 4H-SiC crystals. Mater. Sci. Forum.

[34] Borysiuk J., Sołtys J., Bożek R., Piechota J., Krukowski S., Strupiński W., Baranowski J.M., Stępniewski R. (2012). Role of structure of C-terminated 4H-SiC(000$\bar{1}$) surface in growth of graphene layers: Transmission electron microscopy and density functional theory studies. Phys. Rev. B.

